# IMPROVING HEALTH AND WELL-BEING OF GRANDPARENTS RAISING GRANDCHILDREN: A STRENGTHS-BASED INTERVENTION

**DOI:** 10.1093/geroni/igz038.1292

**Published:** 2019-11-08

**Authors:** Christine Fruhauf, Loriena Yancura, Aimee Fox, Nathaniel Riggs, Heather Greenwood-Junkiermeyer, A N Mendoza

**Affiliations:** 1 Colorado State University, Fort Collins, Colorado, United States; 2 University of Hawaii at Manoa, Manoa, Hawaii, United States; 3 The Ohio State University, Columbus, Ohio, United States

## Abstract

Many grandparents raising grandchildren experience depression. Few interventions take a strengths-based approach to improve their mental health. To address this gap, this study utilized an adapted version of Powerful Tools for Caregivers (PTC) for grandparents (PTC-G) to improve their self-care, communication, and self-efficacy. Grandparents completed self-assessments including the CES-D short form prior to the intervention, immediately after the 6-week program, and at 6-months. Focus groups were also conducted during the 6-month follow-up to further explore positive behavior change. Data from all sources were analyzed to show that the PTC-G program significantly lowered depressive symptoms of grandparents raising grandchildren. Qualitative data shows that grandparents report increased awareness and use of self-care practices and community services. By improving the health and well-being of grandparents raising grandchildren, the PTC-G intervention shows promise in reducing depression and improving long-term mental health outcomes in vulnerable grandfamilies.

